# Identification of 22q11.2 deletion in a patient with schizophrenia and clinically diagnosed Rubinstein–Taybi syndrome

**DOI:** 10.1002/pcn5.34

**Published:** 2022-07-28

**Authors:** Yasuhito Nagai, Masaki Nishioka, Tatsuki Tanaka, Takahisa Shimano, Eiji Kirino, Toshihito Suzuki, Tadafumi Kato

**Affiliations:** ^1^ Department of Psychiatry Juntendo University School of Medicine Tokyo Japan; ^2^ Department of Psychiatry Juntendo Tokyo Koto Geriatric Medical Center Tokyo Japan; ^3^ Department of Psychiatry Juntendo University Hospital Tokyo Japan; ^4^ Department of Psychiatry Juntendo University Koshigaya Hospital Koshigaya Japan; ^5^ Department of Psychiatry Nakamura Hospital Yoshikawa Japan; ^6^ Department of Psychiatry Juntendo University Shizuoka Hospital Izunokuni Japan

**Keywords:** 22q11.2 deletion syndrome, autism spectrum disorder, *HERC1*, Rubinstein–Taybi syndrome, schizophrenia

## Abstract

**Background:**

Rubinstein–Taybi syndrome (RTS) is a rare autosomal‐dominant disease. Almost all cases are sporadic and attributed to de novo variant. Psychotic symptoms in RTS are rare and have been reported in only a few published cases. On the other hand, 22q11.2 deletion syndrome is the most common chromosomal microdeletion in humans. The 22q11.2 deletion is well recognized as a risk factor for schizophrenia. Here, we present a schizophrenic psychosis case clinically diagnosed as RTS but resolved as carrying 22q11.2 deletion by genomic analysis.

**Case presentation:**

A 38‐year‐old Japanese male was admitted to our hospital due to psychotic symptoms. He had been diagnosed with RTS based on physical characteristics at the age of 9 months. On admission, we performed whole exome sequencing. He had no pathogenic variant in *CREBBP* or *EP300*. We detected 2.5 Mb deletion on 22q11.2 and one rare loss‐of‐function variant in a loss‐of‐function‐constrained gene (*MTSS1*) and three rare missense variants in missense‐constrained genes (*CELSR3*, *HERC1*, and *TLN1*). Psychotic symptoms were ameliorated by the treatment of risperidone.

**Conclusion:**

The psychiatric manifestation and genomic analysis may be a clue to detecting 22q11.2 deletion syndrome in undiagnosed patients. The reason for similarity in physical characteristics in 22q11.2 deletion syndrome and RTS remains unresolved. The 22q11.2 deletion and *HERC1* contribute to the patient's phenotype.

## Graphical abstract

Venn diagram for explanation of the overlap phenotypes.

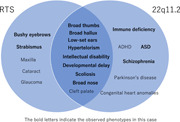

## BACKGROUND

Rubinstein–Taybi syndrome (RTS) is a rare autosomal‐dominant disease. Almost all cases are sporadic and attributed to de novo variant. The male‐to‐female ratio of RTS is almost even, and the incidence is 1:100,000 to 1:125,000. The condition is diagnosed based on clinical symptoms, including distinctive facial features; broad, short thumbs; large toes; microcephaly; scoliosis; reduced growth; and intellectual disability.^1^ The majority of RTS patients are found to have pathogenic variants of *CREBBP* (50%–70%) on chromosome 16p13.3, whereas some cases have the pathogenic variants of *EP300* (5%–8%), located on 22q13.2, but no genetic variants are found in 22%–45% of RTS patients.^1^ Although behavioral symptoms stemming from intellectual disability are common in RTS, to our knowledge, psychotic symptoms in RTS are rare and have been reported in only a few published cases, without genetic analysis.^2^, ^3^ On the other hand, 22q11.2 deletion syndrome (22q11.2 DS) is the most common chromosomal microdeletion in humans. The 22q11.2 DS is caused by hemizygous deletion of the long arm of chromosome 22. The estimated prevalence is one in 4000 live births. In about 90% of the syndrome, the deletion is a de novo occurrence. In the remaining 10%, the deletion is inherited from one of the parents. The 22q11.2 DS often accounts for neurodevelopmental and neurodegenerative disorders: autism spectrum disorder, attention deficit hyperactivity disorder, intellectual disability, schizophrenia, and Parkinson's disease. About 85%–90% of 22q11.2 DS cases have a 3‐Mb deletion, and the remaining 5% have only 1.5‐Mb proximal deletion.^4^ Here, we present a schizophrenic psychosis case clinically diagnosed as RTS but resolved as carrying 22q11.2 deletion by genomic analysis.

## CASE PRESENTATION

A 38‐year‐old Japanese male was admitted to our hospital due to psychotic symptoms. His height was 165 cm, following childhood growth hormone therapy, and his weight was 76 kg. Past medical history included diabetes mellitus, hyperuricemia, hypothyroidism (due to the adverse effect of ^131^I therapy), and strabismus. His physical features included broad thumbs and toes, low‐set ears, hypertelorism, bushy eyebrows, broad nose, and mild scoliosis, which are all typical in RTS.

He was born on term as the second of two siblings, with a birth weight of 3360 g. From 3 months to 3 years of age, he was admitted to hospitals to treat pneumonia three times a year because of his immune deficiency. At the time, he manifested epilepsy with abnormal electroencephalography records. When he reached the age of 9 months, he was diagnosed with RTS based on physical characteristics without genetic analysis. His first words were at 1.5 years of age, but he could not walk without holding onto something, and he used a walker until he entered elementary school. At age 3 years, he spoke in two‐word sentences. With age, he began to show developmental delay, including intellectual disability, deficits in social interaction, impaired communication skills, and repetitive behavior. After graduating from high school and vocational school, he worked as a shop assistant but made many mistakes and did not work well.

At age 22, he showed his first clinical manifestation of psychotic symptoms: auditory hallucinations and delusions of persecution. He had withdrawn socially, and by the time he was 26, his hallucinations and delusions had intensified. He was irritable and aggressive without emotional context. He was diagnosed with schizophrenia according to DSM‐IV criteria. With 600 mg/day of sultopride and risperidone 2 mg/day, he achieved remission, and sultopride was tapered off at discharge. However, the delusions remained and because he still complained of anxiety compulsively, the risperidone was continued.

**Figure 1:**
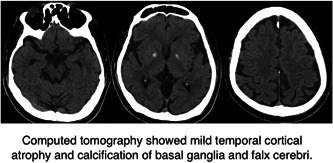
Computed tomography showed mild temporal cortical atrophy and calcification of basal ganglia and falx cerebri.

**Figure 2:**
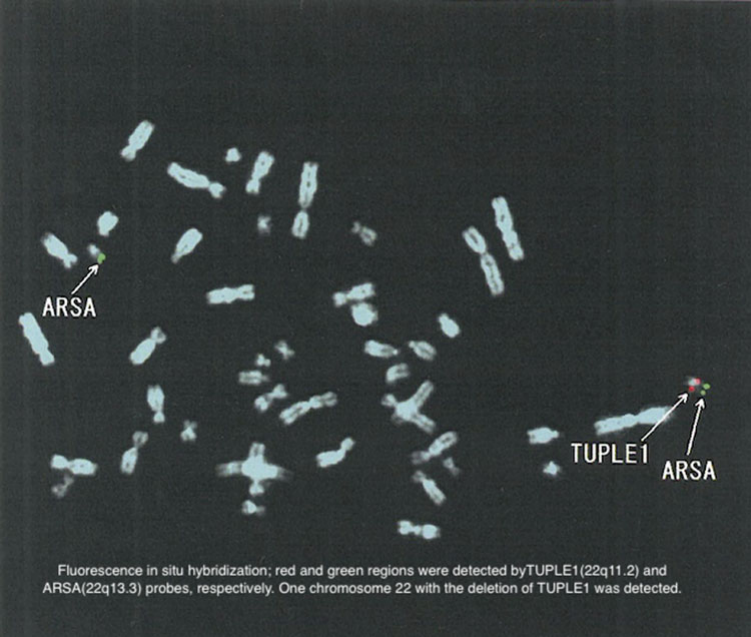
Fluorescence in situ hybridization; red and green regions were detected by TUPLE1(22q11.2) and ARSA(22q13.3) probes, respectively. One chromosome 22 with the deletion of TUPLE1 was detected.

At age 38, behavioral abnormalities reappeared, including wandering around his home, and talking meaninglessly. He reported three persons in his brain and that someone influenced his body. He was admitted to the hospital and found to have a recurrence of auditory hallucinations and delusions of persecution, along with incoherent thinking. On examinations, computed tomography showed mild temporal cortical atrophy and calcification of basal ganglia and falx cerebri (Figure 1). Cytogenetic analysis showed a normal karyotype (46, XY). Whole‐exome sequencing (WES) showed a probably damaging missense variant in *CREBBP* (p.Leu551Ile), but this variant is not rare in East Asians with an allele frequency of more than 1% (gnomAD r2.1.1, https://gnomad.broadinstitute.org/). No other potentially pathogenic variants were found in *CREBBP* or *EP300*. Thus, we searched for variants with potential pathogenic effects unbiasedly (see Supporting Information). We found probable 22q.11.2 deletion by WES, which was confirmed by fluorescence in situ hybridization (Figure 2), and one rare loss‐of‐function (LoF) variant in an LoF‐constrained gene (*MTSS1*) and three rare missense variants in missense‐constrained genes (*CELSR3*, *HERC1*, and *TLN1*). The deletion was further confirmed by array comparative genomic hybridization (SurePrint G3 Human CGH 1x1M Microarray [Agilent]) as a deletion in chr22:18,894,339‐21,464,119 in the GRCh37 coordinate.

The patient's IQ (Wechsler Adult Intelligence Scale 4th Edition) was 47. According to the DSM‐5, he was diagnosed with 22q11.2 DS, autism spectrum disorder, moderate intellectual disability, and schizophrenia. He was prescribed risperidone 2 mg/day, later increasing it to 6 mg/day. Two months after admission, his psychotic symptoms were ameliorated, and the Positive and Negative Syndrome Scale score decreased by 21%. However, he showed anxiety and fear of leaving the hospital, and the hospitalization was prolonged.

## DISCUSSION

**Figure 3:**
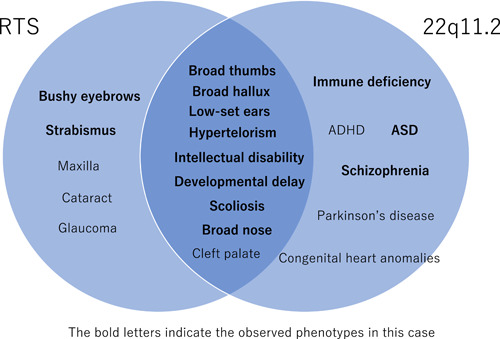
The overlap phenotypes between RTS and 22q11.2 DS. The bold letters indicate the observed phenotypes in this case.

The p‐Leu551Ile variant of *CREBBP* found in this patient was previously reported by Schorry et al.^5^ in a RTS patient. However, its pathological significance has been questioned, and it is now classified as a neutral variant.^6^ In this case, a variant causative for RTS could not be identified in *CREBBP* or *EP300* by the exome sequencing. Therefore, we performed an unbiased genomic analysis and could diagnose this patient as actually having 22q11.2 deletion. The 22q11.2 DS is often suspected in the presence of congenital cardiovascular anomalies in early life. Congenital heart defects have been reported in 70% of all cases. However, the prevalence decreases with the patient's age and is reported in less than 30% of adult cases.^7^ Like previous Japanese cases of 22q11.2DS with schizophrenic psychosis, our patient has mental retardation, no congenital heart defects^8^, ^9^, ^10^, ^11^ and basal ganglia calcification.^9^, ^10^, ^11^ Although he presented several typical symptoms of the syndrome, his distinctive physical characteristics made his diagnosis very difficult. Patients with 22q11.2 DS are known to exhibit physical characteristics similar to RTS^1^, ^9^, ^12^; our case had broad thumbs and hallux, low‐set ears, bushy eyebrows, hypertelorism, broad nose, scoliosis, developmental delay, and intellectual disability. The clinical diagnosis of RTS is straightforward in typical cases^13^ at least for specialists of genetic diseases in children, based on these physical signs. Indeed, according to PubCaseFinder (https://pubcasefinder.dbcls.jp/?lang=en), a site to identify genetic diseases by characteristic signs, the top candidate disease suggested by these signs was RTS, and 22q11.2 DS was not included in the top 10 candidates. Thus, the diagnosis of RTS is unquestionable in this case. However, these signs can also be seen in 22q11.2 DS (Figure 3). Thus, we can interpret that this case is the overlap of RTS and 22q11.2 DS.

The 22q11.2 deletion is a pleiotropy for neuropsychiatry phenotype: schizophrenic symptoms and intellectual disability. The autistic aspect of this patient should be caused by 22q11.2 deletion.^4^ However, 22q11.2 deletion is not fully penetrant for schizophrenia. Common variants associated with schizophrenia could aggregately contribute to schizophrenic symptoms in 22q11.2 DS patients.^14^, ^15^ We speculate that rare damaging variants in this case, in which the pathological effects are uncertain, could also contribute to the psychiatric symptoms. *HERC1* is associated with intellectual disability^16^, ^17^, ^18^, ^19^ and schizophrenia, while the association derives mainly from LoF variants.^20^ From our experience, the phenotype‐based diagnosis is challenging, and the comprehensive genomic analysis, including exome sequencing, should be helpful for the differential diagnosis and understanding of the genetic architecture of rare syndrome with psychiatric symptoms. Although most medical institutions do not have access to WES, comparative genome hybridization analysis for both RTS and 22q11.2 DS has been covered by health insurance in Japan since October 2021. Early diagnosis of 22q11.2 DS is important for the medical care of the patients because we can predict and manage somatic symptoms based on the diagnosis. Age‐related change manifestations and phenotype diversity of 22q11.2 DS make a diagnosis difficult. Many patients with 22q11.2 DS remain undiagnosed until adulthood due to the absence of major physical phenotypic hallmarks. The psychiatric manifestation and genomic analysis may be a clue to detecting 22q11.2 DS in undiagnosed patients.

## CONCLUSION

The 22q11.2 deletion and *HERC1* contributed to our patient's phenotype. However, the reason for similarity in physical characteristics in 22q11.2 deletion syndrome and RTS remains unresolved. We hope that this report contributes to further investigations.

## AUTHOR CONTRIBUTIONS

Yasuhito Nagai, Takahisa Shimano, and Tatsuki Tanaka treated the patient. Masaki Nishioka was involved in genetic analysis. Yasuhito Nagai, Masaki Nishioka, Eiji Kirino, and Tadafumi Kato were involved in data interpretation. All authors participated in discussion, writing this manuscript, and revisions. All read and approved the final version of the manuscript.

## CONFLICT OF INTEREST

The authors declare no conflict of interest.

## ETHICS APPROVAL STATEMENT

This report was approved for publication by the ethics committee of Juntendo University Koshigaya Hospital.

## PATIENT CONSENT STATEMENT

The patient and his mother provided written informed consent to have genetic screenings and publication of this case.

## Supporting information

Supporting information.

## Data Availability

The data that support the findings of this report are available in the supplementary information files.
