# Supplementary material for: Identification of 22q11.2 deletion in a patient with schizophrenia and clinically diagnosed Rubinstein–Taybi syndrome
Source: PCN Rep. 2022 Jul 28;1(3):e34. doi: 10.1002/pcn5.34 (PMC11114328; doi:10.1002/pcn5.34)
Supplement: Supplementary file 1 — Supporting information. [file PCN5-1-e34-s001.docx]

**Supplementary Information**

**Whole exome sequencing**

We extracted genomic DNA from the patient's blood with the standard phenol-chloroform protocol. The exonic regions were enriched with the Agilent SureSelect Human All Exon v6 kit (Agilent Technologies, Santa Clara, CA, USA) according to the manufacturer's instructions. The prepared DNA libraries underwent sequencing by HiSeq2000 (Illumina, San Diego, CA, USA) with paired-end 110bp reads.

**Variant detection and annotation**

We performed read alignment and variant calling according to the Best Practice workflow of the GATK ^1, 2^ (https://gatk.broadinstitute.org/hc/en-us). In brief, the raw sequence reads were aligned to the reference genome hg38 by BWA-0.7.17 ^3^ mem algorithm. The aligned data (bam file format) underwent deduplication with Picard-2.24.0 and base quality score recalibration with GATK-4.1.0.0. We called the short variants (single nucleotide variants [SNVs] and short insertions/deletions) with GATK-4.1.0.0 HaplotypeCaller by single sample mode and selected the variants with the following properties: GQ > 90, SOR < 3, MQ > 55, QD > 5.

The Detected variants in coding regions (N = 23,863) were annotated with the following information: the effect on protein function predicted by SnpEff-4.3 ^4^, allele frequencies in gnomAD r2.1.1 in non-neuro samples (N = 104,068) (gnomAD.non_neuro.AF) ^5^, allele frequencies in ToMMo 8.3K JPN (ToMMo.AF) ^6^, and the predicted effect of missense variants on protein function by MPC from ExAC database (ExAC.MPC) ^7^, M-CAP ^8^ and PrimateAI ^9^ as widely-used computational prediction using machine learning on large datasets. The genes with detected variants were annotated with pLI (probability of loss-of-function intolerance) score and missense Z score in gnomAD r2.1.1 database (gnomAD.pLI and gnomAD.missense.Zscore, respectively).

We called the copy number variations (CNVs) by XHMM-1.0 ^10^ with in-house exome sequencing data derived from exon capturing with the same kit (i.e., Agilent SureSelect Human All Exon v6 kit) and selected the called regions with the following properties: Q_SOME > 90 and length > 500 kb. The reliable CNV was only chr22:19038865-21055176 deletion (22q11.2 microdeletion) in this case.

**Selection of rare variants with potentially damaging effect**

We selected rare variants with potentially damaging effects on the protein functions with computational prediction. We first filtered out the variants frequently observed in large genomic panels (gnomAD.nonneuro.AF > 0.001 or ToMMo.AF > 0.001) and homozygous variants as those tolerating natural selection. We next selected damaging variants as follows: (i) loss-of-function (LoF) variants in LoF-intolerant genes (defined as gnomAD.pLI > 0.90), (ii) missense variants with MPC > 1 in missense-intolerant genes (defined as missense Z score in gnomAD > 4.0). This procedure resulted in one LoF and three missense variants potentially damaging the protein functions.

| GENE | *MTSS1* | *HERC1* | *CELSR3* | *TLN1* |
| --- | --- | --- | --- | --- |
| Variant type | frameshift | missense | missense | missense |
| HGVS.p | p.Ser759fs | p.Asp4803Asn | p.Arg1862Trp | p.Ala2059Thr |
| ExAC.MPC | N/A | 1.07 | 1.88 | 1.38 |
| gnomAD.non_neuro.AF | 1.92 × 10^-5^ | 4.96 × 10^-6^ | 2.90 × 10^-5^ | 3.85 × 10^-5^ |
| ToMMo.AF | 0.0003 | 0.0003 | 0.0001 | 0.0007 |
| gnomAD.pLI | 0.9997 | 1.000 | 1.000 | 1.000 |
| gnomAD.missense.Zscore | 1.81 | 4.96 | 4.08 | 4.67 |
| M-CAP | N/A | Damaging | Damaging | Tolerated |
| PrimateAI | N/A | Damaging | Tolerant | Tolerant |

*The variant information is limited to amino acid changes to protect the patient's privacy.

The missense variant in *HERC1* is also predicted to be damaging both with M-CAP and PrimateAI, while the allele frequency indicates that this variant is not ultra-rare in the Japanese population (ToMMo.AF = 0.003). However, the amino acid of Asp4803 (D4803) and around D4803 is well conserved across various vertebrates (figure below, from USSC Genome Browser: http://genome.ucsc.edu/), which suggests that the domain including D4803 is functionally important in the HERC1 protein.


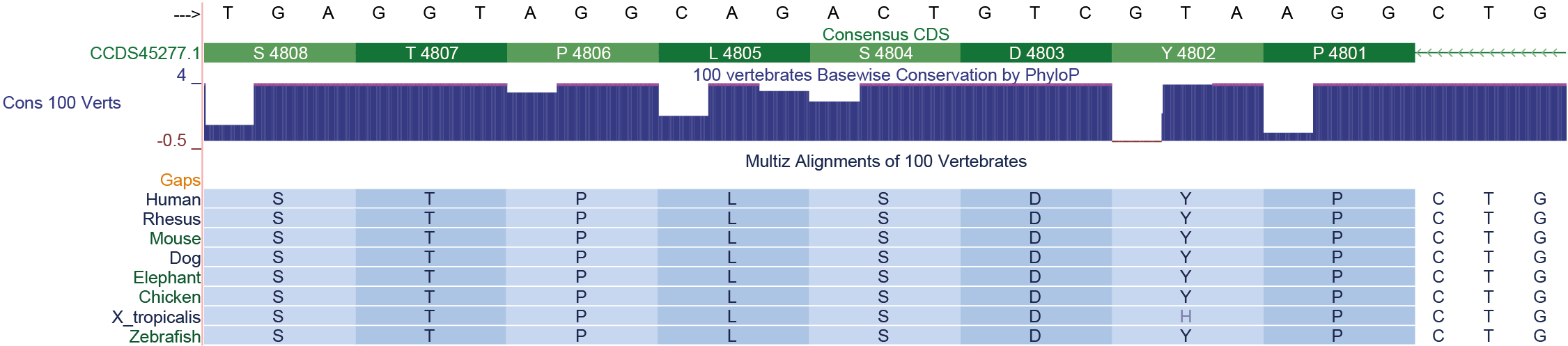


**Supplementary Reference**

1. McKenna A, Hanna M, Banks E et al. The Genome Analysis Toolkit: a MapReduce framework for analyzing next-generation DNA sequencing data. *Genome Res* 2010; **20**: 1297-303.

2. DePristo MA, Banks E, Poplin R et al. A framework for variation discovery and genotyping using next-generation DNA sequencing data. *Nat Genet* 2011; **43**: 491-8.

3. Li H, Durbin R. Fast and accurate short read alignment with Burrows-Wheeler transform. *Bioinformatics* 2009; **25**: 1754-60.

4. Cingolani P, Platts A, Wang le L et al. A program for annotating and predicting the effects of single nucleotide polymorphisms, SnpEff: SNPs in the genome of Drosophila melanogaster strain w1118; iso-2; iso-3. *Fly (Austin)* 2012; **6**: 80-92.

5. Karczewski KJ, Francioli LC, Tiao G et al. The mutational constraint spectrum quantified from variation in 141,456 humans. *Nature* 2020; **581**: 434-443.

6. Nagasaki M, Yasuda J, Katsuoka F et al. Rare variant discovery by deep whole-genome sequencing of 1,070 Japanese individuals. *Nat Commun* 2015; **6**: 8018.

7. Samocha KE, Kosmicki JA, Karczewski KJ et al. Regional missense constraint improves variant deleteriousness prediction. *bioRxiv* 2017: 148353.

8. Jagadeesh KA, Wenger AM, Berger MJ et al. M-CAP eliminates a majority of variants of uncertain significance in clinical exomes at high sensitivity. *Nat Genet* 2016; **48**: 1581-1586.

9. Sundaram L, Gao H, Padigepati SR et al. Predicting the clinical impact of human mutation with deep neural networks. *Nat Genet* 2018; **50**: 1161-1170.

10. Fromer M, Moran JL, Chambert K et al. Discovery and statistical genotyping of copy-number variation from whole-exome sequencing depth. *Am J Hum Genet* 2012; **91**: 597-607.
